# Electrophysiological behavior of neonatal astrocytes in hippocampal *stratum radiatum*

**DOI:** 10.1186/s13041-016-0213-7

**Published:** 2016-03-22

**Authors:** Shiying Zhong, Yixing Du, Conrad M. Kiyoshi, Baofeng Ma, Catherine C. Alford, Qi Wang, Yongjie Yang, Xueyuan Liu, Min Zhou

**Affiliations:** Department of Neurology, Shanghai 10th Hospital of Tongji University, School of Medicine, 301 Yan Chang Zhong Road, Shanghai, 200072 China; Department of Neuroscience, Ohio State University Wexner Medical Center, Columbus, OH 43210 USA; Department of Neuroscience, Tufts University School of Medicine, Boston, MA USA

**Keywords:** Astrocytes, Hippocampus, K^+^ conductance, K^+^ homeostasis, Gap junctions

## Abstract

**Background:**

Neonatal astrocytes are diverse in origin, and undergo dramatic change in gene expression, morphological differentiation and  syncytial networking throughout development. Neonatal astrocytes also play multifaceted roles in neuronal circuitry establishment. However, the extent to which neonatal astrocytes differ from their counterparts in the adult brain remains unknown.

**Results:**

Based on ALDH1L1-eGFP expression or sulforhodamine 101 staining, neonatal astrocytes at postnatal day 1–3 can be reliably identified in hippocampal *stratum radiatum*. They exhibit a more negative resting membrane potential (*V*_M_), −85 mV, than mature astrocytes, −80 mV and a variably rectifying whole-cell current profile due to complex expression of voltage-gated outward transient K^+^ (*I*K_a_), delayed rectifying K^+^ (*I*K_d_) and inward K^+^ (*I*K_in_) conductances. Differing from NG2 glia, depolarization-induced inward Na^+^ currents (*I*Na) could not be detected in neonatal astrocytes. A quasi-physiological *V*_M_ of −69 mV was retained when inwardly rectifying K_ir_4.1 was inhibited by 100 μM Ba^2+^ in both wild type and TWIK-1/TREK-1 double gene knockout astrocytes, indicating expression of additional leak K^+^ channels yet unknown. In dual patch recording, electrical coupling was detected in 74 % (14/19 pairs) of neonatal astrocytes with largely variable coupling coefficients. The increasing gap junction coupling progressively masked the rectifying K^+^ conductances to account for an increasing number of linear voltage-to-current relationship passive astrocytes (PAs). Gap junction inhibition, by 100 μM meclofenamic acid, substantially reduced membrane conductance and converted all the neonatal PAs to variably rectifying astrocytes. The low density expression of leak K^+^ conductance in neonatal astrocytes corresponded  to a ~50 % less K^+^ uptake capacity compared to adult astrocytes.

**Conclusions:**

Neonatal astrocytes predominantly express a variety of rectifying K^+^ conductances, form discrete cell-to-cell gap junction coupling and are deficient in K^+^ homeostatic capacity.

## Background

Neonatal astrocytes have been traditionally viewed as immature astrocytes undergoing extensive changes in cell proliferation, establishment  of spatially distinct domains, integration into syncytial network through gap junction coupling, wrapping of blood vessels as part of the blood brain barrier, and varying in gene expression to reach functional maturity [1–8]. Emerging evidence shows that neonatal astrocytes also play a pivotal role in synaptogenesis and facilitate myelination that is essential for neuronal circuit wiring and brain function [8–10]. In view of the critical role of neonatal astrocytes in developing brain, it becomes important to know the basic functional properties and how they behave electrophysiologically in their early life.

The first wave of astrogliogenesis peaks around E20-P3 in various regions of the rodent brain, and astrocytes in postnatal days 1–3 should mainly arise from direct transformation of ventricular zone (VZ) radial glia and asymmetric division of glial progenitor cells [11–17]. In contrast, after a short dormant period [4], the second wave of astrogliogenesis mainly produces astrocytes through symmetric division of differentiated astrocytes and to a less extent asymmetric division of NG2 glia [5, 18]. However, to what extent the newborn  astrocytes from the two distinct phases differ in their electrophysiological properties is poorly defined. In the present study, we focused on neonatal astrocytes in the P1-3 dormant period and asked the following questions. First, whether neonatal astrocytes, deriving from the first wave of astrogliogenesis, in the hippocampus share markers which commonly appear in mature astrocytes, such as GFAP, chemical marker sulforhodamine 101(SR101) and gene marker ALDH1L1 [6, 19, 20], as shown in P2 spinal cord astrocytes [12, 21]. Second, whether the diversity in astrocytic origins corresponds to heterogeneity in electrophysiological properties. Third, whether neonatal astrocytes are electrophysiologically distinct compared to proliferating astrocytes in postnatal brain and mature astrocytes. Fourth, whether neonatal astrocytes are strongly electrically coupled as has been observed in the adult brain. Information from this critical early developmental stage is essential for our further understanding of the role of neonatal astrocytes in the developing brain.

By taking advantage of ALDH1L1-eGFP transgenic mouse and SR101 as live cell markers for identification of newborn astrocytes in P1-3 *stratum radiatum*, we show that neonatal astrocytes are electrophysiologically characterized by a more negative resting membrane potential and a homogeneous expression of a distinct set of rectifying K^+^ channels. In contrast with mature astrocytes [22], neonatal astrocytes form discrete electrical coupling early on in postnatal life. Furthermore, neonatal astrocytes are much less capable of redistributing K^+^ ions across the membrane. These unique features should have profound implications for the complex roles of neonatal astrocytes in the developing brain.

## Methods

### Animals

All the experimental procedures were performed in accordance with a protocol approved by the Animal Care and Use Committees of The Ohio State University. The wild type C57BL/6J and BAC-ALDH1L1-eGFP transgenic mice were used in the present study [23], as well as TWIK-1/TREK-1 double gene knockout mice [24]. Neonatal hippocampal astrocytes from postnatal day (P) 1–3 mice of both sexes were used.

### Preparation of acute hippocampal slices

Hippocampal slices were prepared as described previously. Briefly, brains were rapidly removed from skulls and placed into ice-cold oxygenated (95 % O_2_/5 % CO_2_) slice cutting aCSF with reduced Ca^2+^ and increased Mg^2+^ (in mM): 125 NaCl, 3.5 KCl, 25 NaHCO_3_, 1.25 NaH_2_PO_4_, 0.1 CaCl_2_, 3 MgCl_2_ and 10 Glucose. Coronal hippocampal slices (300 μm) were cut at 4 °C with a Vibratome (Pelco 1500) and transferred to the oxygenated standard aCSF (in mM): 125 NaCl, 25 NaHCO_3_, 1.25 NaH_2_PO_4_, 3.5 KCl, 2 CaCl_2_, 1 MgCl_2_ and 10 Glucose_,_ osmolality, 295 ± 5 mOsm; pH 7.3–7.4), recovering at room temperature for at least one h before recording or Sulforhodamine 101 (SR101) incubation (see below).

### Fresh dissociation of single hippocampal astrocytes

As we described previously in detail [25, 26], coronal hippocampal slices at 250 μm thickness were sectioned from P21–25 mice and incubated in oxygenated aCSF. One to three slices were transferred from standard aCSF to oxygenated Ca^2+^-free aCSF at 34 °C supplemented with 0.6 μM astrocytic marker SR-101 for 30 min. After incubation, the CA1 regions were dissected out from slices, cut into small pieces (1 mm^2^), and transferred into a 1.5 ml Eppendorf tube containing oxygenated aCSF supplemented with 24U/ml papain and 0.8 mg/ml *L*-cysteine for incubation for 7 min at 25 °C. The loosened tissues after papain digestion were gently triturated 5–7 times into a cell suspension, and transferred into the recording chamber mounted on the microscope. Although the cell suspensions contain multiple tissue blocks, only single dissociated astrocytes were used in this study [26].

### Sulforhodamine 101 staining

For sulforhodamine 101 (SR101) [20], the slices were transferred to a slice-holding basket containing 0.6 μM SR101 in aCSF at 34 °C for 30 min. Then, the basket was transferred back to normal aCSF at room temperature before the experiment. Some of the slices from BAC-ALDH1L1-eGFP transgenic mice were mounted immediately after SR101 staining to analyze the colocalization of SR101 and ALDH1L1-eGFP in CA1 *stratum radiatum* region using a confocal microscope (LSM510, Carl Zeiss).

### Imaging acquisition

A fluorescent imaging system, Polychrome V system (Till Photonics, Germany), was used for identification of astrocytes from ALDH1L1-eGFP or SR101 staining neonatal astrocytes in slices. This system was also used for high resolution visualization of small glial soma for whole-cell astrocyte recording [27].

### Immunohistochemistry

The hippocampal slices were fixed in 4 % paraformaldehyde for 1 h (h) at room temperature. Permeabilization was then followed in 0.2 % Triton X-100 PBS for 1 h. The slices were then incubated with a blocking solution consisting of 5 % normal donkey serum (DNS) and 0.01 % Triton X-100 in PBS for 3 h. The primary anti-GFAP antibody, goat anti-GFAP (1:1000, Abcam, Cambridge, MA), was diluted into a 10 % DNS/0.005 % Triton X-100 solution and applied to slices at 4 °C overnight. Following rise of slices with blocking solution, the secondary antibody, Alex555 donkey anti-goat (1:1000), was applied for 1 h at room temperature. Immunofluorescence images were obtained from a confocal microscope (LSM510, Carl Zeiss). To reliably identify colocalization of GFAP immunostaining signal with eGFP in ALDH1L1-eGFP mice, only the cellular somas showing ALDH1L1-eGFP alone, or together with GFAP staining signal, were selected in this analysis.

### Electrophysiology

For brain slice recording, individual hippocampal slices were transferred to the recording chamber mounted on an Olympus BX51WI microscope, with constant perfusion of oxygenated aCSF (2.0 ml/min). Astrocytes located in the *stratum radiatum* region were visualized using an infrared differential interference contrast (IR-DIC) video camera. Whole-cell patch clamp recordings were performed using a MultiClamp 700A amplifier and pClamp 9.2 software (Molecular Devices, Sunnyvale, CA). Borosilicate glass pipettes (Warner Instrument, Hamden, CT) were pulled from a Micropipette Puller (Model P-87, Sutter Instrument). The recording electrodes had a resistance of 2–5 MΩ when filled with the electrode solution containing (in mM) 140 KCl, 13.4 NaCl, 0.5 CaCl_2_, 1.0 MgCl_2_, 5 EGTA, 10 HEPES, 3 Mg-ATP, and 0.3 Na-GTP (280 ± 5 mOsm, PH 7.25–7.35). To examine K^+^ uptake capacity, the intracellular K^+^ was fully substituted with Na^+^ ions.

The membrane potential (*V*_M_) was recorded under current clamp mode in PClamp 9.2 program. The liquid junction potential was compensated prior to forming the cell-attached mode for all recordings. In current clamp recording, the input resistance (*R*_in_) was measured by “Resistance test” protocol in PClamp 9.2 software (pulse: 63 pA/600 ms) before and after recording. When R_in_ varied greater than 10 % during recording, the cells were discarded. In recordings where voltage clamping quality was significantly improved after inhibition of gap junction coupling, the access resistance (*R*_a_), membrane resistance (*R*_M_) and membrane capacitance (C_M_) were measured from “Membrane test” protocol available in PClamp 9.2 software. Also, only those recordings which achieved an initial *R*_a_ less than 15 MΩ and varied less than 10 % were included in data analysis. All the experiments were conducted at room temperature.

### Chemical reagents

SR101 was purchased from Invitrogen (New York, NY). All other chemicals and salts used in intracellular and extracellular solutions were purchased from Sigma-Aldrich. 100 μM BaCl_2_ and the 100 μM meclofenamic acid (MFA) were dissolved directly in aCSF.

### Data analyses

To calculate coupling coefficient (CC) and determine the rectification characteristic of gap junctions in neonatal astrocytes, the stimulated cell (*C*_stim._) in dual patch recording was set in voltage clamp mode, and transjunctional voltage (*V*_transjunction_) in the recipient cell (*C*_reci._) was measured in zero holding current clamp mode. The CC between the recording pair at each command voltage step (*V*_COM_) was calculated from:

$$ \mathrm{C}\mathrm{C}\left(\%\right)=\Delta {V}_{M, Creci.}/\Delta {V}_{M, Cstim.}\times 100\% $$

Where the ∆*V*_M_ in *C*_stim._ was calculated from ∆*V*_M_ = ∆I_M_×(*R*_t_-*R*_a_). The ∆I_M_ was measured in the end of each *V*_COM_ step. *R*_a_, access resistance, *R*_t_, total resistance.

The intracellular K^+^ concentrations ([K^+^]_i_s) were calculated from the Goldman-Hodgkin-Katz equation in the following form:

$$ E=\mathrm{R}\mathrm{T}/\mathrm{F}\mathrm{l}\mathrm{n}\left(\left({P}_{\mathrm{K}}{\left[{\mathrm{K}}^{+}\right]}_{\mathrm{e}}+{P}_{\mathrm{Na}}{\left[\mathrm{N}{\mathrm{a}}^{+}\right]}_{\mathrm{e}}+{P}_{\mathrm{Cl}}{\left[\mathrm{C}{\mathrm{l}}^{-}\right]}_{\mathrm{i}}\right)/\left({P}_{\mathrm{K}}{\left[{\mathrm{K}}^{+}\right]}_{\mathrm{i}}+{P}_{\mathrm{Na}}{\left[\mathrm{N}{\mathrm{a}}^{+}\right]}_{\mathrm{i}}+{P}_{\mathrm{Cl}}{\left[\mathrm{C}{\mathrm{l}}^{-}\right]}_{\mathrm{e}}\right)\right) $$

[*x*]_i_ and [*x*]_e_ are referred intracellular and extracellular ion concentrations, respectively. For astrocytes, the *P*_K_ is 1, *P*_Cl_ is assumed at 0 and *P*_Na_ is 0.015 [28].

The patch clamp recording data were analyzed by Clampfit 9.0 (Molecular Devices, Sunnyvale, CA) and Origin 8.0 (OriginLab, Northhampton, MA). Results are given as mean ± SEM. Statistical analysis was performed using one-way ANOVA. Significance level was set at *P* <0.05.

## Results

### Identification of neonatal astrocytes in hippocampal *stratum radiatum*

Lack of astrocytic stage-specific markers remains a challenge for the lineage tracing of astrocytes in embryonic and neonatal stages. ALDH1L1 emerged as a highly expressed protein in astrocytes from gene expression profiling and has also been demonstrated to be an early and reliable gene marker for identification of ALDH1L1-expression cells from embryonic day (E) 9.5 onward [6, 10, 23, 29]. In the present study, BAC-ALDH1L1-eGFP transgenic mice were used to identify neonatal astrocytes in the hippocampal *stratum radiatum* region. We found that eGFP-expression cells in ALDH1L1-eGFP mice were always morphologically correlated with glial cells characterized by a soma size < 10 μm under the IR-DIC in *stratum radiatum* [23, 25, 30], and none of the recorded eGFP-expression cells turned out to be excitable neurons in patch clamp recording.

**Fig. 1:**
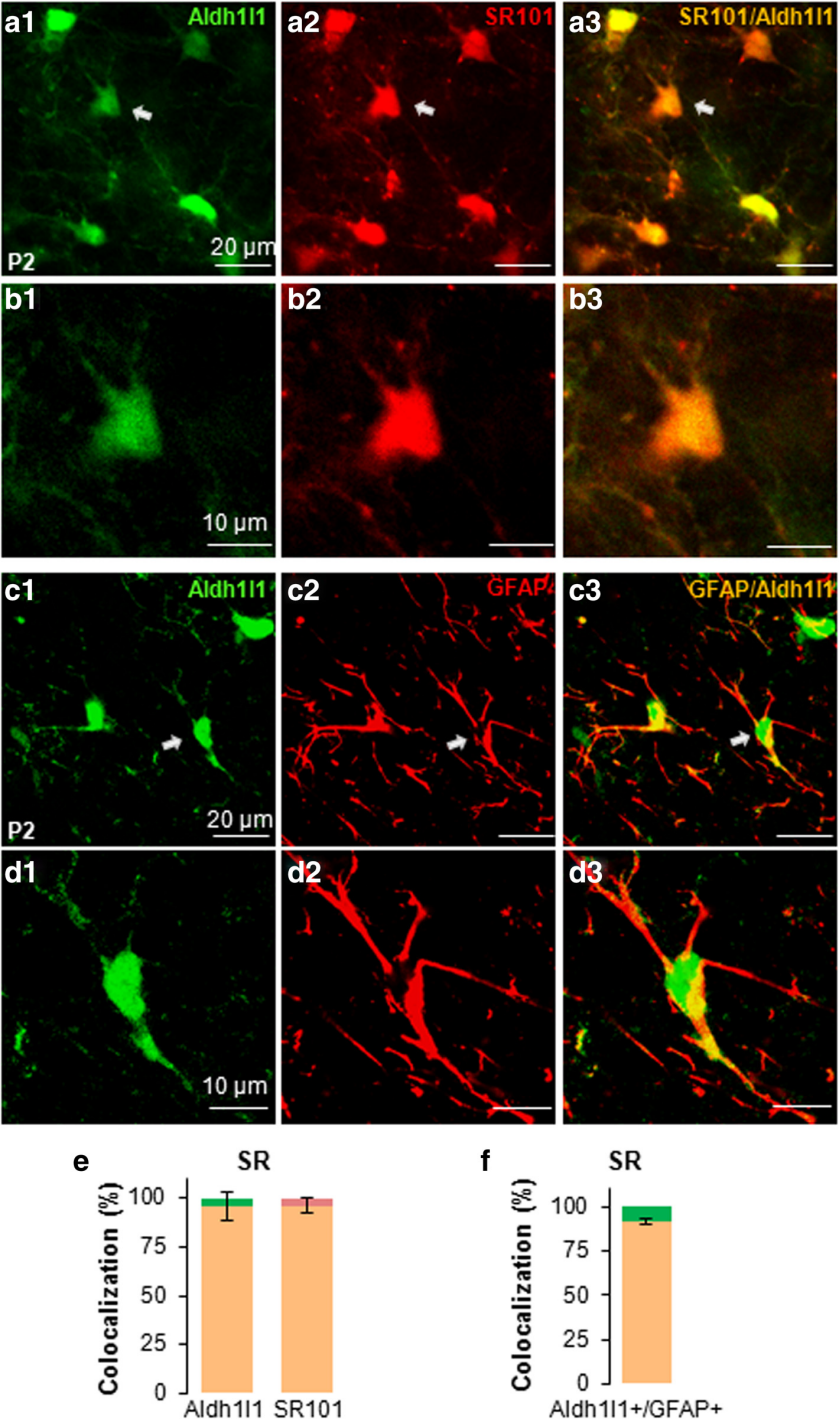
Identification of neonatal astrocytes in hippocampal *stratum radiatum* region. Neonatal astrocytes in *stratum radiatum* region revealed in BAC-ALDH1L1-eGFP mice (**a1**). The same ALDH1L1-eGFP^+^ cells in **a1** were co-localized with SR101 staining (**a2**–**3**). High magnification images of an ALDH1L1-eGFP^+^/SR101 stained cell, indicated by arrows in **a1**–**3**, are shown in **b1**–**3**. The eGFP and SR101 staining show a similar subcellular labeling pattern. The eGFP in the somas of neonatal astrocytes identified in ALDH1L1-eGFP *stratum radiatum* region (**c1**) were co-localized with GFAP immunostaining (**c2**, **c3**). High magnification images of an ALDH1L1-eGFP^+^/GFAP^+^ cell, indicated by arrows in **c1**–**3**, are shown in **d1**–**3**. The difference in subcellular labeling of both markers is evident. **e** The quantification of the colocalization of ALDH1L1-eGFP^+^ and SR101. **f** The quantification of the colocalization of ALDH1L1-eGFP^+^ and GFAP

We next examined the colocalization of ALDH1L1-eGFP^+^ cells with another astrocytic marker SR101 [20] (Fig. 1a, b and e). 96.2 ± 3.9 % of SR101^+^ cells were ALDH1L1-EGFP^+^ (*n* =547, 3 animals). Likewise, 96.3 ± 7.5 % ALDH1L1-EGFP^+^ cells were also SR101^+^ (*n* =541, 3 animals) (Fig. 1e). To further confirm an astrocytic identify of ALDH1L1-EGFP^+^ cells, co-localization of ALDH1L1-eGFP^+^ cells with GFAP immunostaining signal, a classic astrocytic marker, was examined [10, 19, 31] (Fig. 1c, d and f). 91.5 ± 1.7 % of ALDH1L1-eGFP^+^ cells showed GFAP positive immunostaining (*n* = 266, Fig. 1f). Thus, both ALDH1L1-eGFP and SR101 are reliable and specific markers for identification of neonatal astrocytes in *stratum radiatum* region, and these markers were used in the following functional study.

### Neonatal astrocytes exhibit two distinct electrophysiological phenotypes

**Fig. 2:**
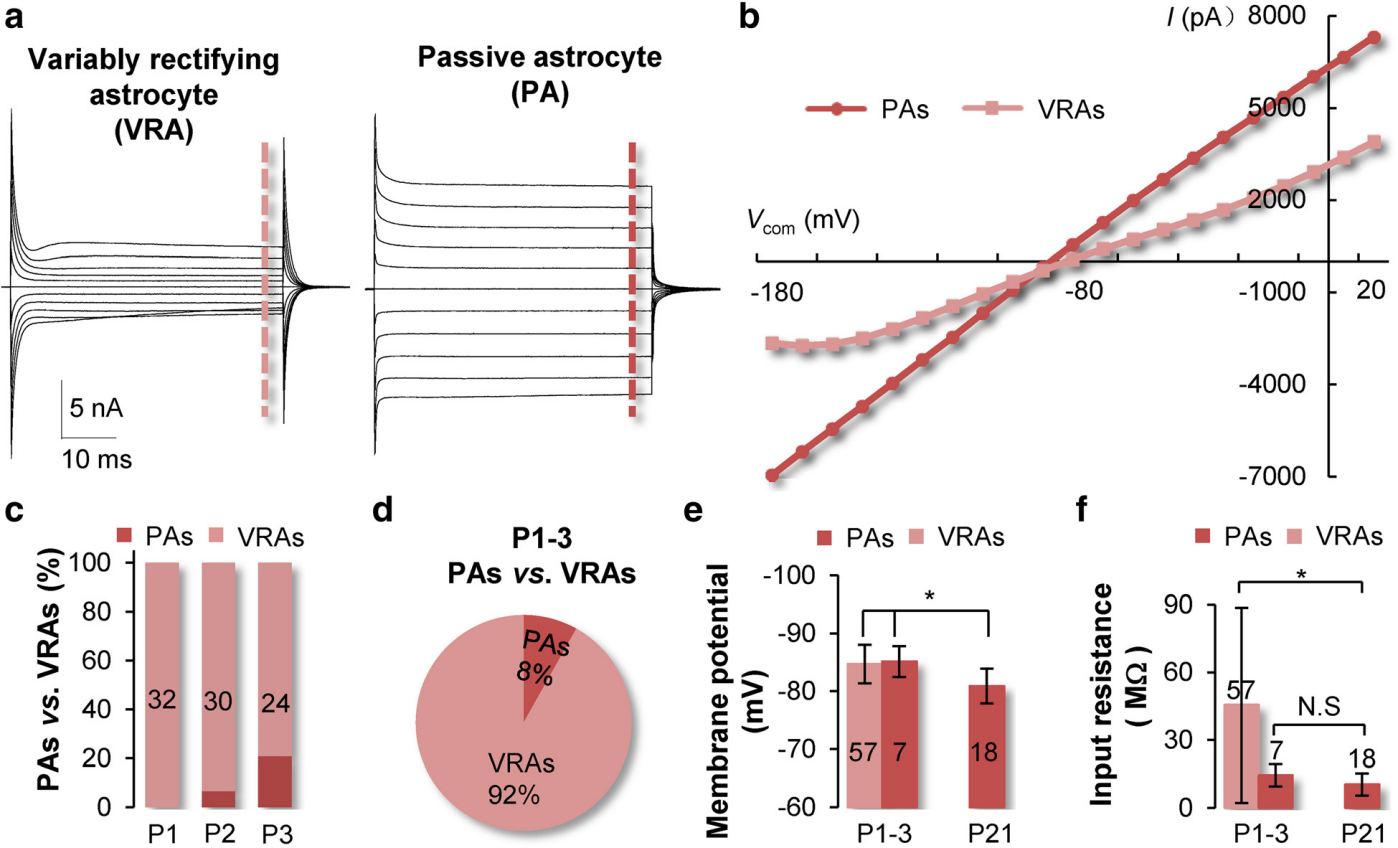
Neonatal astrocytes exhibit two distinct electrophysiological phenotypes. **a** Two whole-cell voltage clamp recordings from a P1 (left) and a P3 (right) neonatal astrocyte, respectively. The cells were held at −80 mV at resting, and the 50 m voltage commands (*V*_COM_) were from −180 to +20 mV with 10 mV increments. **b** The variably rectifying astrocytes (VRA) show multiple rectification points in I-V plot, whereas the I-V plot in passive astrocytes (PA) was rather linear. **c** PAs first appeared in P2 (6.67 %) and increased to 20.83 % in P3. **d** The percentages of VRAs and PAs in P1-3 *stratum radiatum* region. **e** The *V*_M_ values in VRAs and PAs are comparable in neonatal astrocytes, but are significantly more negative than those of astrocytes > P21. **f** The R_in_ varied markedly in VRAs and was significantly higher than PAs in both neonatal and adult animals.*: *P* < 0.05. N.S, *P* > 0.05

Three electrophysiological phenotypes of astroglia, i.e., astrocytes and NG2 glia, have been described in previous reports [2, 32]. However, a systematic analysis of neonatal astrocytes could not be done, because a reliable marker for identification of live astrocytes for functional study was not available at the time. By taking advantage of ALDH1L1-eGFP mouse and SR101 staining *in situ*, two distinct electrophysiological phenotypes were observed in *stratum radiatum* region from P1 to 3 mice (Fig. 2a). In whole-cell voltage clamp recording, the P1 neonatal astrocytes identically showed a variably rectifying astrocyte (VRA) current profile, characterized by expression of voltage-gated outward transient K^+^ (*I*K_a_), delayed rectifying K^+^ (*I*K_d_) and inward K^+^ (*I*K_in_) (*n* = 32, Fig. 2a, b, further details in Fig. 5). The second phenotype, passive astrocyte (PA), characterized by a linear current-to-voltage (*I-V*) relationship whole-cell conductance, emerged from P2 (Fig. 2a, b), and the number of PAs increased from 6.67 % in P2 (*n* = 30) to 20.83 % at P3 (*n* = 24) (Fig. 2c). Overall, VRAs and PAs amounted to 92 and 8 % of recorded neonatal astrocytes (*n* = 86), respectively (Fig. 2d). Both VRAs and PAs showed comparable membrane potential (*V*_M_): −84.7 ± 3.3 mV in VRAs (*n* = 57), vs. −85.1 ± 2.7 mV (*n* = 7) in PAs (*P* > 0.05). Noticeably, the *V*_M_ of neonatal astrocytes was significantly more negative than mature PAs in P21 and older animals, −80.9 ± 3.0 mV (*n* = 18, *P* < 0.05) (Fig. 2e). The membrane input resistance (R_in_) varied substantially in VRAs, 45.4 ± 43.2 MΩ (*n* = 57), and was significantly higher than PAs in neonatal (14.3 ± 4.9 MΩ, *n* = 7) and mature (10.3 ± 4.9 MΩ, *n* = 18) animals (*P* < 0.05, Fig. 2f).

### Gap junction coupling masks the activation kinetics of intrinsic rectifying K^+^ conductances

**Fig. 3:**
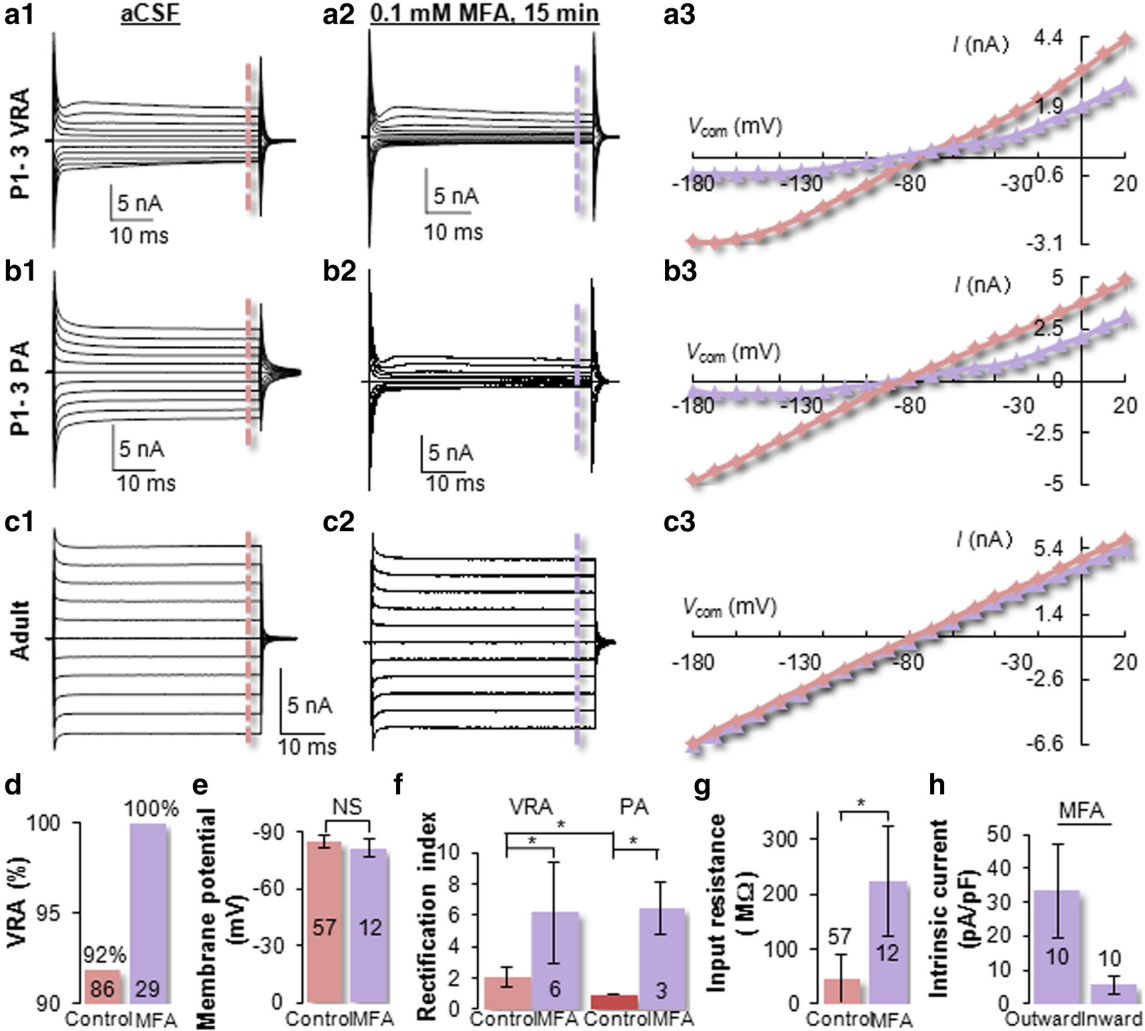
Gap junction coupling masks the activation kinetics of intrinsic ion channels in neonatal astrocytes. **a**, **b** An initially identified VRA (**A1**) and a PA (**B1**) were followed by a 15 min 100 μM MFA bath perfusion for gap junction coupling inhibition. A substantial reduction in overall whole-cell conductance, especially the inward conductance, occurred in both cells (**A2**, **B2**). Remarkably, MFA converted the PA to VRA. **A3** and **B3** illustrate the *I-V* plots of MFA effects in VRA and PA. **c** A PA from a P21 mouse was subjected to the same MFA treatment, but did not show change in electrophysiological phenotype (**C3**). **d** MFA does not affect the *V*_M_ of neonatal astrocytes. **e** In the presence of MFA, the electrophysiological PA phenotype was completely absent in neonatal astrocytes. **f** The whole-cell rectification index (RI) was significantly increased in both VRAs and PAs. **g** The overall R_in_ of neonatal astrocytes was significantly increased in the presence of MFA. **h** In the presence of MFA, the intrinsic ionic currents can be accurately determined. The current density (pA/pF) of outward current, at the steady-state level, was more than 6-folds higher than that of the inward currents. *. *P* < 0.05. N.S, *P* > 0.05

Gap junction coupling has been shown to obscure the activation kinetics of intrinsic ionic conductances in olfactory ensheathing cells (OECs), GFAP-expressing cells of the postnatal subventricular zone, and P9 astrocytes [33–35]. To determine whether cell-to-cell coupling accounts for the passive behavior of neonatal astrocytes, 100 μM meclofenamic acids (MFA), a gap junction inhibitor, was bath applied for 15 min after initial identification of electrophysiological phenotypes. This substantially reduced membrane conductance in both VRAs and PAs (Fig. 3a, b), but not the passive conductance of mature astrocytes (*n* = 6, Fig. 3c). Interestingly, MFA altered all the initially identified PAs to VRAs (*n* = 3) (Fig. 3b). In a separate set of experiments with 1 h pre-incubation of P3 hippocampal slices with 100 μM MFA, all the neonatal astrocytes identically showed a VRA phenotype (*n* = 29, Fig. 3d). These experiments demonstrated that gap junction coupling is causal for the passive behavior of neonatal astrocytes.

Consistent with our previous observation that MFA does not affect *V*_M_ and passive conductance in mature astrocytes [3, 22, 36], the *V*_M_ in neonatal astrocytes was unchanged between control (−84.70 ± 3.35 mV, *n* = 57) and MFA (−83.19 ± 6.22 mV, *n* = 12, *P* > 0.05, Fig. 3e). To determine how coupling affects the activation of intrinsic ion channels in neonatal astrocytes, the rectification index (RI) was intruduced in analysis [37]. The RI increased by 3- and 6-folds in VRAs (2.02 ± 0.64 in control vs. 6.18 ± 3.21 in MFA, *n* = 6, *P* < 0.05) and PAs (0.96 ± 0.02 in control vs. 6.44 ± 1.63 in MFA, *n* = 3, *P* < 0.05), respectively (Fig. 3f). After 100 μM MFA treatment, the R_in_ in neonatal VRA increased from 42.0 ± 41.9 MΩ (*n* = 57) to 223.7 ± 100.3 MΩ (*n* = 12) (*P* < 0.05, Fig. 3g). Under this uncoupled condition, the intrinsic K^+^ conductances could be accurately quantified. The outward and inward steady-state currents were 33.2 ± 14.1 pA/pF, and 5.5 ± 2.7 pA/pF (*n* =10), respectively (Fig. 3h).

In summary, neonatal astrocytes predominantly express rectifying K^+^ conductances and are electrophysiologically homogeneous. Additionally, a developmental increase in gap junction coupling progressively masks the activation of rectifying K^+^ conductances that underlies the passive behavior of neonatal astrocytes.

### Neonatal astrocytes predominantly express rectifying K^+^ conductances

Previously, depolarization-induced inward Na^+^(*I*Na), outward transient (*I*K_a_) and delayed rectifying (*I*K_d_) conductances, and hyperpolarization-induced inward K^+^(*I*K_in_) conductances were described in neonatal astroglia [38, 39]. Now the availability of reliable markers for live astrocyte identification and a better voltage-clamping quality achieved through MFA-induced uncoupling allows examination of rectifying K^+^ conductances in neonatal astrocytes with high fidelity.

**Fig. 4:**
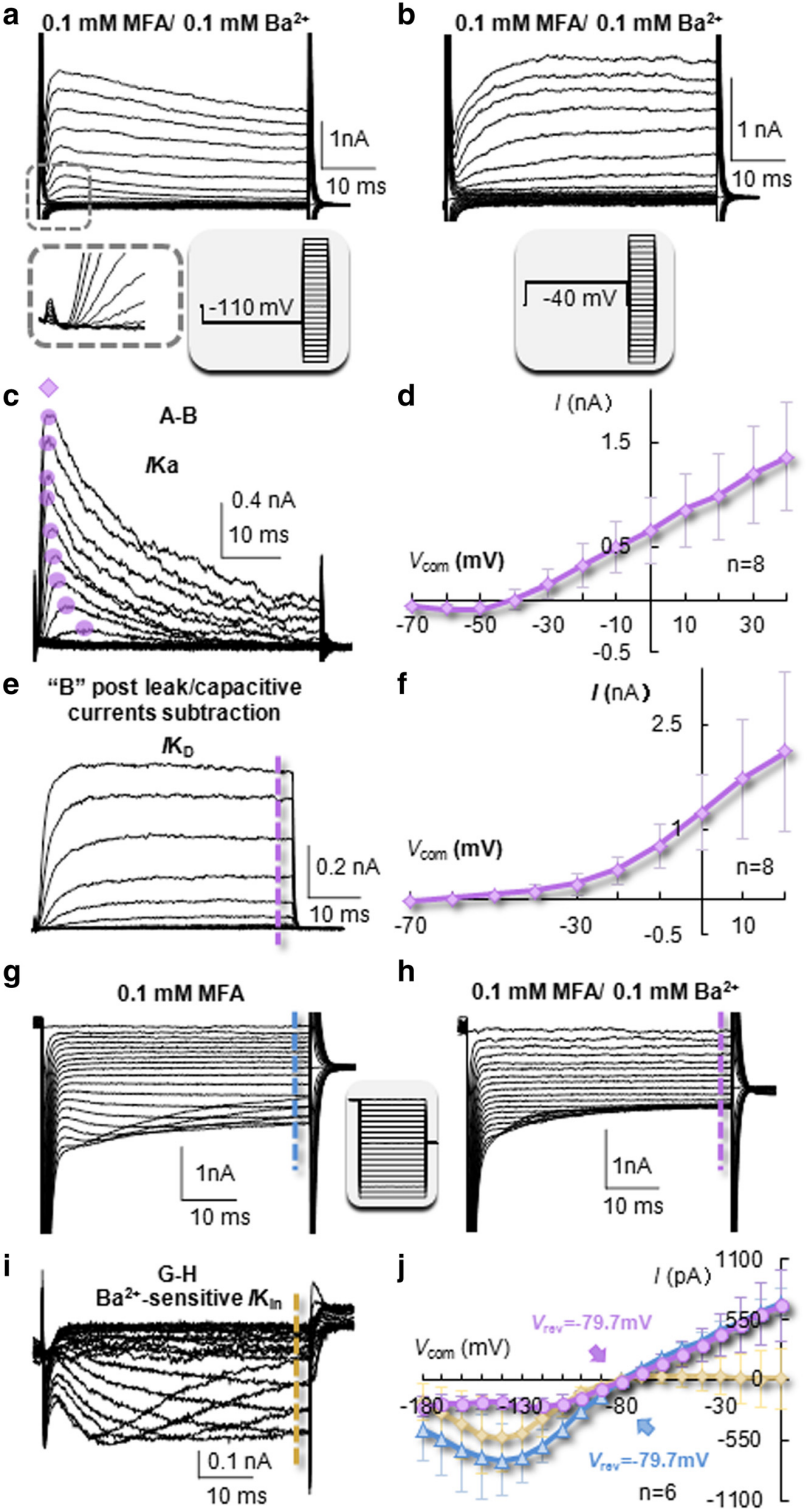
Complex expression of rectifying K^+^ conductances in neonatal astrocytes. **a** To maximally induce outward *I*K_a_, and potential inward *I*Na, a −110 mV/300 ms prepulse was followed by a series of 50 ms steps from −180 to +40 mV with 10 mV increments. Absence of any depolarization-induced inward *I*Na was shown in the inset below (**a**) after symmetric subtraction of leak and capacitive currents. **b** To eliminate *I*K_a_, a prepulse at −40 mV/300 ms was followed by test steps from −180 to +40 mV with 10 mV increments and 50 ms duration. **c**, **d** The isolated *I*K_a_ and its corresponding I-V plot with a *V*_rev_ at −40 mV. The *I*K_a_ was isolated by off-line subtraction of recording (**a** and **b**). **e**, **f** Isolated *I*K_d_ and its corresponding I-V plot. The *I*K_d_ was isolated by symmetric leak and capacitive current subtraction from recording (**b**). **g** Inward K^+^ conductance (*I*K_in_) in a P1 astrocyte induced after inhibition of gap junction coupling by incubation of slice with 100 μM MFA. For *I*K_in_ activation, a 500 ms pre-pulse at 0 mV was followed by a set of steps from −180 to 0 mV, 50 ms duration and 10 mV increments. **h** Addition of 100 μM Ba^2+^ for 15 min substantially reduced the initial *I*K_in_. **i** Subtracted Ba^2+^-sensitive currents. **j** I-V plots illustrate the total *I*K_in_, Ba^2+^-sensitive *I*K_in_ and the remaining outward Ba^2+^-insensitive currents as indicated by color codes. The Ba^2+^ -sensitive and insensitive conductances were reversed at −79.7 mV and 79.7 mV, respectively

Under uncoupled conditions in MFA, the depolarization induced *I*K_a_, *I*K_d_ and potential *I*Na can be isolated based on their different biophysical properties [40]. Specifically, *I*K_a_ and potential *I*Na could be maximally induced by using a −110 mV/300 ms prepulse preceding the test voltages (inset in Fig. 4a), whereas inactivation of the same K^+^ and Na^+^ conductances could be achieved by adding a −40 mV/300 ms prepulse prior to the test voltages (inset in Fig. 4b) [38]. The latter voltage protocol allowed selective activation of *I*K_d_. Whole-cell currents resulting from these command protocols are shown in Fig. 4a and b, respectively. Notably, in recording using the first protocol for maximal *I*Na activation (Fig. 4a), the symmetric leak and capacitive current subtraction resulted in no detectable *I*Na in all neonatal astrocytes (inset in Fig. 4a, *n* = 91), which differed from our previously reported *I*Na in NG2 glia recorded under the same conditions [39]. The *I*K_a_ was isolated by digital current subtraction of whole-cell currents recorded from the two voltage protocols as shown in Fig. 4c, and the resulted I-V plot showed a characteristic voltage- and time-dependent activation with a reversal potential (*V*_rev_) of −39.5 ± 8.7 mV (*n* = 8) (Fig. 4d). After symmetric leak and capacitive current subtraction for whole-cell currents recorded from the second voltage protocol (Fig. 4b), the resulted *I*K_d_ and its corresponding I-V plot characteristically showed a voltage-dependent and delayed activation of outward K^+^ conductance with a *V*_rev_ of −61.4 ± 10.4 mV (*n* = 8) (Fig. 4e, f). Overall, neonatal astrocytes predominantly express voltage-gated *I*K_a_ and *I*K_d_, but do not show detectable voltage-gated inward *I*Na.

To inactivate outward K^+^ conductances for selective study of sustained inward K^+^ conductance (*I*K_in_), a 0 mV/500 ms prepulse was delivered prior to test pulses from −180 to 0 mV with 10 mV increments and 50 ms durations [38]. The induced *I*K_in_ showed a characteristic inward rectification and time dependent inactivation of currents at voltages more negative than −140 mV with a whole-cell *V*_rev_ of −79.7 ± 0.8 mV (*n* = 6, Fig. 4g). Addition of 100 μM Ba^2+^ for 10 min substantially reduced the inward currents (Fig. 4h), and the subtracted Ba^2+^-sensitive currents fit well with the activation kinetic of K_ir_4.1 (Fig. 4i) [41]. Interestingly, the remaining Ba^2+^-insensitive currents exhibited a strong outward rectification and still reversed at a quasi-physiological level of −79.1 ± 1.2 mV (*n* = 6), suggesting its identity as a leak K^+^ conductance that follows the GHK constant field rectification (Fig. 4j) [42].

### The identity of Ba^2+^-insensitive leak conductance in neonatal astrocytes

**Fig. 5:**
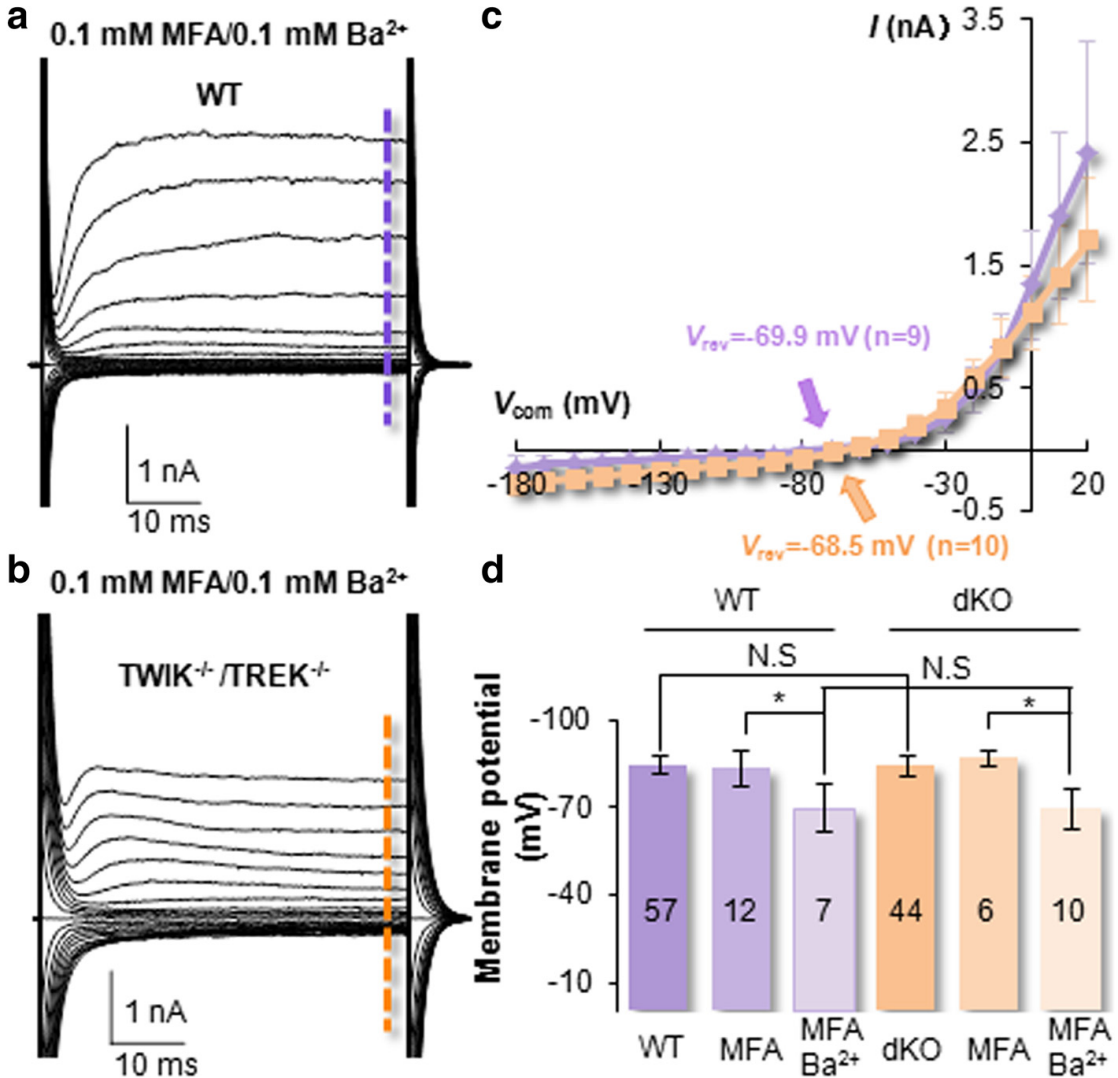
Expression of multiple leak type K^+^ conductances contributing to the resting V_M_ of neonatal astrocytes. **a**, **b**. Whole-cell recording from a P1 wild type and P1 TWIK-1/TREK-1 double gene double knockout (dKO) astrocyte as indicated. Prior to recording, gap junction coupling was inhibited by 100 μM MFA, and K_ir_4.1 was inhibited by 100 μM Ba^2+^. **c** I-V plots of recording (**a** and **b**) as indicated by color codes. The *V*_rev_ was −69.9 and −68.5 mV in recorded WT and dKO astrocytes, respectively. **d** The *V*_M_ values recorded from WT and dKO astrocytes under various conditions indicated. A quasi-physiological *V*_M_ was retained in both WT and dKO astrocytes in the absence of K_ir_4.1 conductance

To explore further the identify of Ba^2+^-insensitive currents that followed GHK outward rectification, we asked whether the GHK rectifying TREK-1 two-pore domain K^+^ channel (K_2p_) would be a potential candidate [6, 24, 43]. Additionally, TWIK-1 K_2p_ is highly expressed in mature astrocytes and its membrane expression is regulated by astrocytic mGluR3 [37, 44]. To explore potential contribution of these K_2p_s to the remaining Ba^2+^-insensitive currents, the TWIK-1/TREK-1 double gene knockout mouse (dKO) was used in the following experiment [24]. Under the conditions that gap junction coupling and K_ir_4.1 were inhibited, the remaining whole-cell currents in dKO astrocytes showed a similarly strong outward rectification as that of WT. Additionally, the steady-state inward currents, activated at −180 mV voltage step, still amounted to −139.5 ± 90.4 pA (*n* = 9) in WT (Fig. 5a, b) and −279.8 ± 95.1 pA (*n* = 10) in dKO neonatal astrocytes (*P* < 0.05) (Fig. 5a, c). Note that in the presence of 100 μM Ba^2+^, a quasi-physiological *V*_M_ remained comparable between WT (−69.9 ± 8.4 mV, *n* = 9) and dKO (−68.5 ± 7.7 mV, *n* = 10) neonatal astrocytes (*P* > 0.05, Fig. 5d). These results indicate that TREK-1 and TWIK-1 contribute minimally to the Ba^2+^-insensitive leak K^+^ conductance, and future study is needed to uncover additional leak K^+^ channels in neonatal and adult astrocytes [45].

### Neonatal astrocytes form discrete cell-to-cell gap junction coupling

**Fig. 6:**
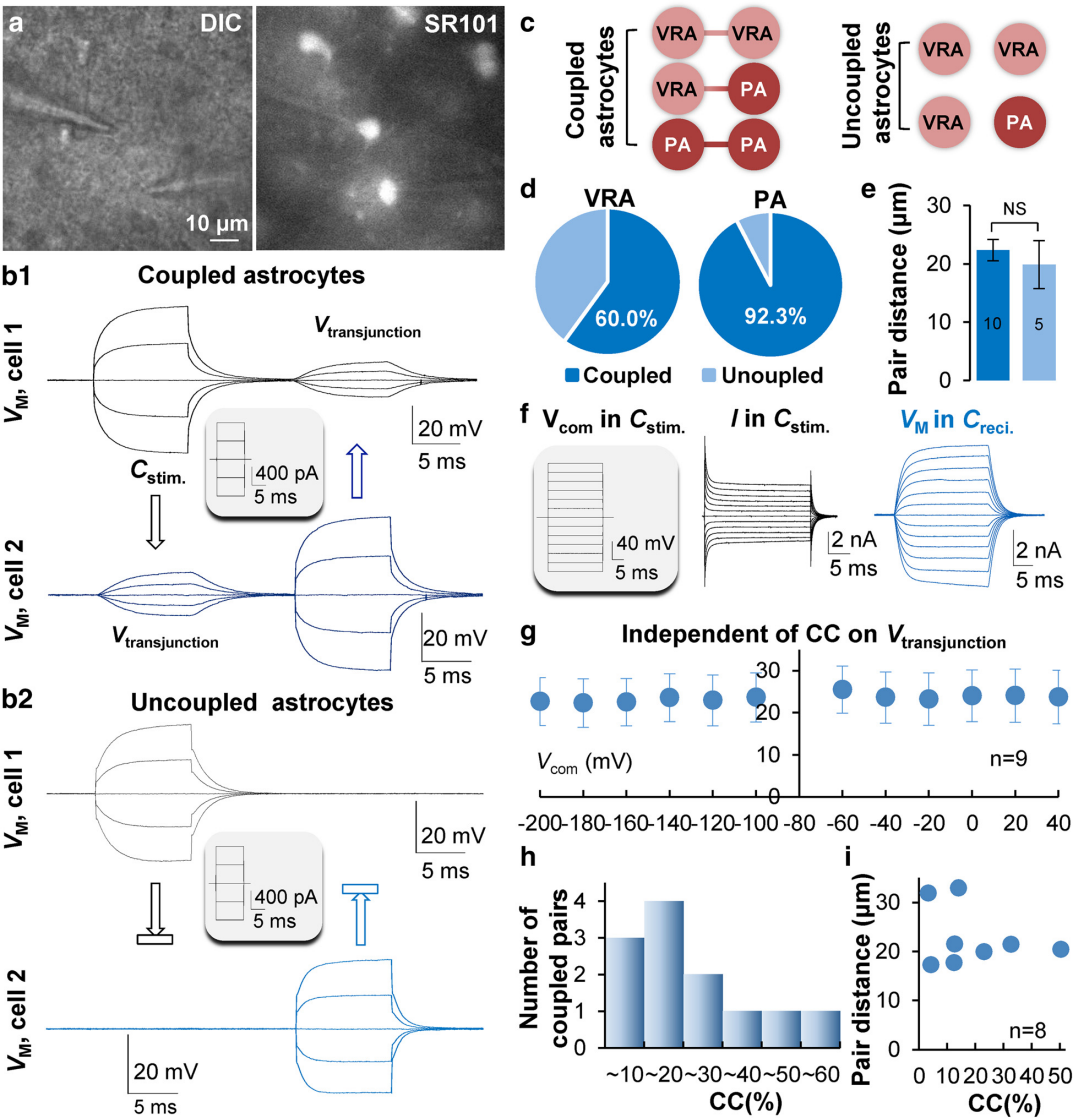
Discrete electrical coupling among neonatal astrocytes. **a** Dual patch recording from a pair of neonatal astrocytes in CA1 *stratum radiatum*. The recorded cells (DIC, left) were selected based on SR101 staining (right). **B1**, **B2**. A pair of electrically coupled (top), and uncoupled astrocytes (bottom), respectively. The current steps, shown in inset, were alternately delivered to the stimulated cell (*C*_stim._) that induced transjunctional voltage (*V*_transjunction_) only in the recipient cell (*C*_reci_.) of the coupled pair (top). **c** The coupling occurred in either VRA-VRA, PA-PA homotypic or VRA-PA heterotypic pairs. Likewise, the uncoupled pairs were not associated with either VRA or PA phenotypes. **d** Nearly all the PAs were coupled compared to 40 % of uncoupled VRAs. **e** The cell-to-cell pair distance was not associated the occurrence of cell coupling. **f** The recording configuration for coupling coefficient (CC) measurement, the *V*_COM_ voltages, ranging from −220 to +60 mV, were delivered to the *C*_stim._ in voltage clamp mode, whereas the *V*_transjunction_ was recorded in the *C*_reci_. in current clamp mode. **g** The CC values varied insignificantly throughout the test *V*_COM_ voltages (*n* = 9, *P* > 0.05). **h** The variation of CC values was independent of cell-to-cell pair distances. **i** The CC varied substantially among all the recorded pairs (*n* = 13)

To gain further insight into gap junction coupling in neonatal astrocytes, dual patch recording was used to determine the electrical coupling between neighboring neonatal astrocytes. The voltages were alternately delivered to one of the cells in a pair, termed the stimulated cell (*C*_stim._), and the induced transjunctional voltages (*V*_transjunction_) were simultaneously recorded from the second cell in a pair, termedthe recipient cell (*C*_reci_.) [22, 36] (Fig. 6a, b). The *V*_transjunction_ could be detected in 14 out of 19 pairs of neonatal astrocytes (73.7 %), and the coupling exhibited in VRA-VRA, PA-PA homotypic or VRA-PA heterotypic pairs (Fig. 6c). Noticeably, PA-PA homotypic pair did show coupling and the percentage of coupled PAs was markedly higher (12/13, 92.3 %) than that of VRAs (15/25, 60 %) (Fig. 6d). Additionally, the coupled and uncoupled pairs did not differ in their cell-to-cell distances (*P* > 0.05, Fig. 6c).

Inhibition of gap junction coupling eliminated the inward conductance more evidently than that of the outward conductance in both VRAs and PAs (Fig. 3a, b). To determine whether a rectifying filter effect is exhibited in gap junction channels to account for this observation, the coupling coefficient (CC) was analyzed over a wide range of voltages from −220 mV to +40 mV (Fig. 6f, Methods in details). The CC values varied only slightly from 22.3 to 25.5 % over the tested voltages (*n* = 9, *P* > 0.05, Fig. 6g), indicating a linear gating property of gap junction channels in neonatal astrocytes. This analysis also showed that the CC values varied markedly among recording pairs (*n* = 13, Fig. 6h), which is independent of the distance between the two patched cells (Fig. 6i).

In view of convergence of neonatal astrocytes from multiple resources, the discrete cell-to-cell coupling in early postnatal life suggests that newly generated astrocytes are uncoupled at birth and the syncytial network should be established progressively in later postnatal development.

### Neonatal astrocytes exhibit a poor K^+^ uptake capacity compared to adult astrocytes

In contrast to mature astrocytes, neonatal astrocytes predominantly express voltage-gated outward K^+^ conductances, whereas the level of leak K^+^ conductance is evidently lower as indicated by their significantly higher membrane input resistances (Fig. 2f). This suggests that neonatal astrocytes should be less efficient in redistributing K^+^ ions across the membrane in the event of change in transmembrane K^+^ driving force [25].

**Fig. 7:**
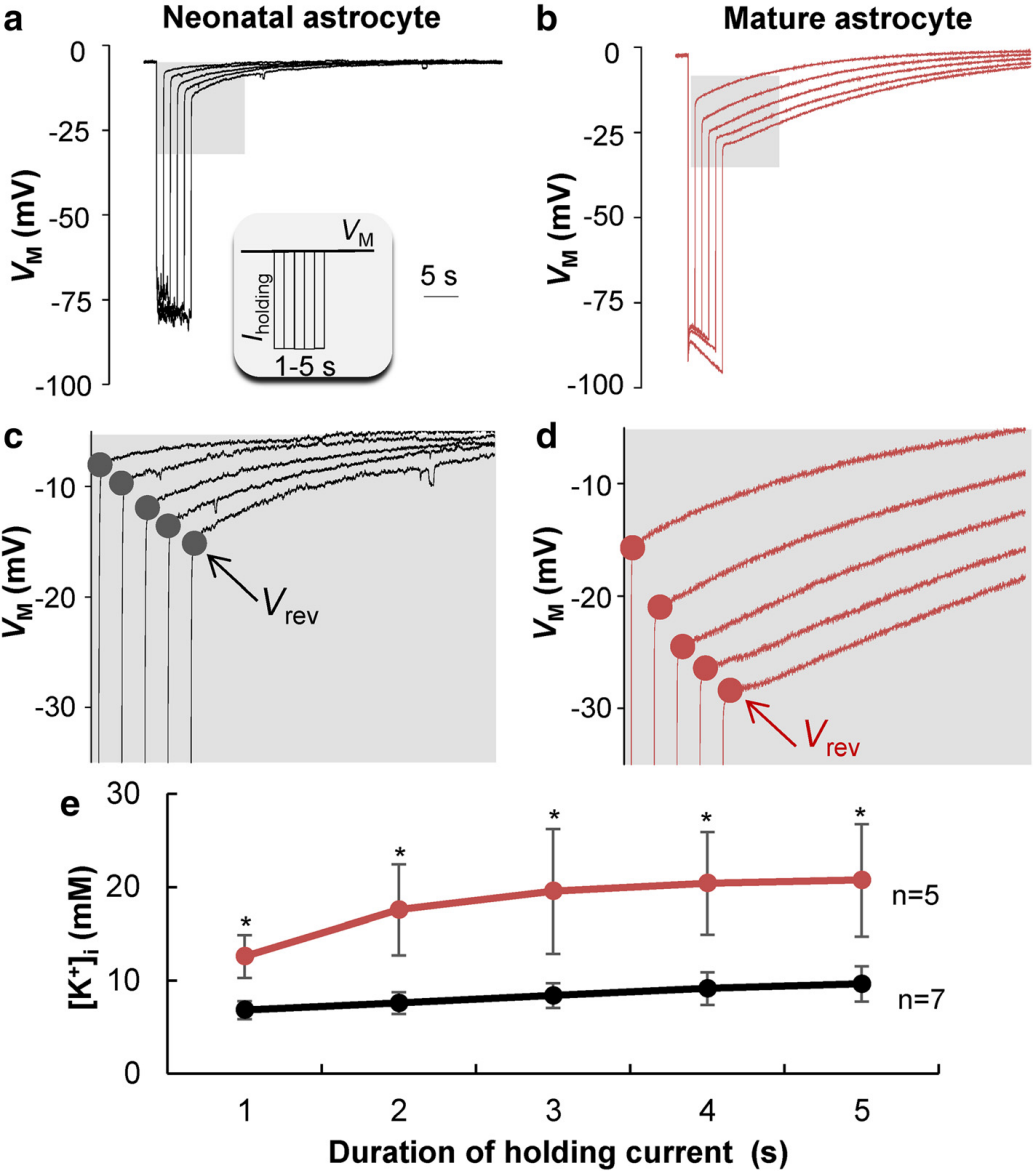
Neonatal astrocytes exhibit a deficient K^+^ uptake capacity. **a**, **b** The *V*_M_ responses from a neonatal and mature astrocyte as indicated. To test K^+^ uptake capacity, the K^+^ ions in the electrode were replaced fully by Na^+^ ions, whereas the bath K^+^ remained at the physiological 3.5 mM. A negative holding current (*I*_holding_) was applied at incremental duration from 1 to 5 s to shift the *V*_M_ downward to −75 mV. In between the *I*_holding_ pulses, the cell was maintained at *I*_holding_ = 0 for *V*_M_ recovery back to the resting levels. The longer the duration of the *I*_holding_ pulses, the larger the induced maximal *V*_M_ hyperpolarization. Also, the longer the duration of *I*_holding_ pulse, the more negative of the *V*_*rev*_s, indicating more accumulation of K^+^ inside astrocytes (see **c** and **d** in expanded scale). The *V*_rev_ deviated much less from the resting *V*_M_ in neonatal astrocytes compared to mature astrocytes. **e** According to GHK equation, the estimated intracellular K^+^ concentrations, corresponding to the *V*_rev_ values, are plotted against the *I*_holding_ pulse durations for neonatal and mature astrocytes as indicated by color codes. Overall, the capacity of K^+^ uptake in neonatal astrocytes is ~50 % less than mature astrocytes

To examine this experimentally, the slices were first pre-incubated with MFA for gap junction inhibition prior to astrocyte recording. In the control experiment, single freshly dissociated hippocampal astrocytes from P21 to 25 mice were used to completely eliminate gap junction coupling [30]. For recording, the intracellular K^+^ was completely substituted by Na^+^ ions as described in our previous study [25]. In current clamp recording, this resulted in a whole-cell *V*_M_ around 0 mV at resting condition (Fig.7a, b). A series of negative holding current (*I*_holding_), duration 1–5 s, was applied to drive *V*_M_ from resting 0 mV to −75 mV (inset in Fig. 7a). The prolonged inward K^+^ drive force resulted in an increasing accumulation of intracellular K^+^ that can be calculated by Goldman-Hodgkin-Katz (GHK) equation from the *V*_*rev*_ values measured immediately after the release of *I*_holding_ pulses. As shown in Fig. 7a, b, the incremental *I*_holding_ pulses induced a duration-dependent negative shift in *V*_rev_ values (shadowed areas) (Fig. 7c, d). According to the GHK calculation, the resulting *V*_rev_ corresponded to an *I*_holding_ duration-dependent increase in intracellular K^+^ concentrations in astrocytes. However, in neonatal astrocytes, 1, 2, 3, 4 and 5 s negative *I*_holding_ resulted in a net intracellular K^+^ accumulation of 6.81 ± 0.95 mM, 7.56 ± 1.17 mM, 8.38 ± 1.33 mM, 9.13 ± 1.76 mM and 9.62 ± 1.90 mM, respectively (*n* = 7) (Fig. 7e). For mature astrocytes, the same experimental conditions resulted in a net intracellular K^+^ accumulation of 12.57 ± 2.30 mM, 17.56 ± 4.88 mM, 19.55 ± 6.69 mM, 20.40 ± 5.51 mM and 20.73 ± 6.01 mM, respectively (*n* = 5) (Fig. 7e). The net intracellular K^+^ accumulation of neonatal astrocytes is significantly lower than that of mature astrocytes (*P* < 0.05). Overall, the K^+^ uptake capacity in neonatal astrocytes is only ~50 % of that of the mature astrocytes.

## Discussion

Increasing evidence suggests that neonatal astrocytes may comprise a unique stage specific population of astrocytes that are multidimensionally involved in postnatal brain development and function. Meanwhile, neonatal astrocytes are diverse in their origins. However, to what extent neonatal astrocytes differ from functionally mature astrocytes, and how their physiological behavior is related to the neonatal brain development and function are questions largely unknown. We show that, compared to mature astrocytes in the same brain region, nascent astrocytes exhibit salient differences in their ion channel expression, gap junction coupling and the ability in regulating the concentration of extracellular K^+^.

### Identification of neonatal astrocytes

A universal marker for identification of astrocytes in the developing and adult brain is still unavailable [10]. In the present study, neonatal astrocytes were identified based on the expression of eGFP in ALDH1L1-eGAP transgenic mice [21, 23] and positive staining to a commonly used chemical marker SR101 [20, 23]. We show that both markers are co-localized well with morphologically identified astro-shaped glial cells in hippocampal *stratum radiatum* [2, 32, 46]. The eGFP-expression cells were nicely co-localized with SR101 stained cells (Fig. 1a), and the eGFP (+) cells were also well co-localized with the gold standard astrocytic marker GFAP (Fig. 1b). None of the identified cells, based on these markers, turned out to be excitable neurons. A majority of the identified cells showed electrical coupling (Fig. 6). Based on these characteristics, the eGFP-expression and SR101 stained neonatal cells satisfied the criterion to be considered astrocytes [47].

It should be noted, however, that the stage-specific and origin-specific markers for differentiating astrocytes with diverse origins, such as radial glia, subventricular zone progenitor cells, NG2 glia and local proliferation remain unavailable [48]. Thus, it is possible that some of the neonatal astrocytes deriving from different sources could potentially be excluded in the present study.

### Neonatal astrocytes are electrophysiologically homogeneous

To better characterize the electrophysiological properties of neonatal astrocytes, we purposely narrowed the animal age to the dormant P1-3 period for examining potential diversity in ion channel expression among neonatal astrocytes. Interestingly, two electrophysiological phenotypes could be readily identified during this early postnatal age. The neonatal astrocytes in P1 homogeneously show a variably rectifying whole cell current profile, whereas electrophysiologically passive astrocytes (PAs) first appear in P2, and the percentage of PAs rapidly increased from 6.67 % in P2 to 20.83 % at P3. Interestingly, the appearance of PA in mice is 2 days earlier than rats [2], which seemingly follows a longer gestation time in rats (22 day) than mice (20 day).

We show that the passive behavior of neonatal astrocytes is solely attributable to gap junction coupling (Fig. 3). This differs fundamentally from the passive behavior of membrane conductance in mature astrocytes that is caused by intrinsic K^+^ channel expression [3, 25, 49, 50]. In our previous studies, MFA was used to inhibit gap junction coupling of mature hippocampal astrocytes that resulted in a 99.3 % of coupling inhibition without altering the passive behavior of membrane conductance, suggesting that MFA-induced transition of PA to VRA was unlikely caused by MFA effect on membrane conductance in neonatal astrocytes.

Several voltage-gated K^+^, Na^+^ and Ca^2+^ conductances have been previously reported to be associated with astro-shaped glia in the early postnatal hippocampus [2, 32, 51–53]. Now we show that neonatal astrocytes predominantly express depolarization-induced outward *I*K_a_ and *I*K_d_. Under uncoupled conditions, the current density (pA/pF) of steady-state outward K^+^ conductance is 6-folds higher than that of the inward (Fig. 3h). This markedly differs from the linear passive conductance in freshly dissociated mature astrocytes [25]. With significantly improved voltage clamp quality in recording, depolarization-induced inward Na^+^ or Ca^2+^ currents were not detectable in neonatal astrocytes (Fig. 4e). Meanwhile, voltage-gated *I*Na has been shown as a characteristic feature of NG2 glia in the developing and mature brain [39, 54, 55]. Thus lack of *I*Na appears to be diagnostic for differentiating astrocytes from NG2 glia.

Although the density of inward K^+^ conductance (*I*K_in_) is substantially lower in neonatal astrocytes, they exhibit a significantly more negative membrane potential (*V*_M_) than mature astrocytes. Furthermore, in the presence of 100 μM Ba^2+^, the remaining Ba^2+^ -insensitive current retained a quasi-physiological *V*_M_ level. Consistent with our recent reports that TWIK-1 and TREK-1 do not contribute to passive conductance and resting *V*_M_, the Ba^2+^ -insensitive currents in TWIK-1/TREK-1 double gene knockout mice remained unchanged. This suggests the presence of additional leak type K^+^ channels contributing to the resting *V*_M_ [45]. A more negative *V*_M_ suggests a further lower Na^+^ permeability in neonatal astrocytes, and a plausible explanation would be a relatively low expression of non-selective cation channels, such as ionotropic P2X, unpaired gap junction hemichannels and TRP channels [56].

In summary, neonatal astrocytes are electrophysiologically homogeneous, characterized by expression of a distinct set of rectifying K^+^ conductances. This ion channel expression profile differs substantially from the passive conductance observed from proliferating astrocytes in the later postnatal developing brain and from mature astrocytes [2, 5].

### Neonatal astrocytes form discrete gap junction coupling

During the postnatal brain development, the number of astrocytes expands 6–8 folds in the postnatal brain [4]. Additionally, in the neonatal brain, astrocytes converge from difference sources [10, 48]. A fundamental question to be answered is whether the nascent astrocytes connect with each other through gap junctions and achieve a syncytial network as mature astrocytes do [30]. To answer this important question, we focused on the newborn astrocytes in *stratum radiatum* to determine how they establish cell-to-cell coupling in their early life. Because we have previously demonstrated that electrical coupling is more sensitive than the dye coupling method [22], electrical coupling was used in the present study to detect gap junction coupling.

In contrast to astrocytes in the adult hippocampus, neonatal astrocytes form discrete cell-to-cell coupling; the electrical coupling was detected in only 74 % of the recorded pairs, suggesting newly produced astrocytes are uncoupled in embryonic and early neonatal brain. Further evidence in support of this notion include the following. First, the percentage of neonatal PAs, resulting from increasing gap junction coupling, increases with age and the electrical coupling was detected from nearly all the PAs (92 %) compared to a substantially low percentage of VRAs (60 %) (Fig. 6). Second, whether the newborn astrocytes show electrical coupling does not depend on their pair distances, and coupling can be formed in homotypic or heterotypic electrophysiological phenotypes (Fig. 6). Third, a substantial variation in coupling coefficient exhibited among recording pairs, and this variation does not show any association with pair distances (Fig. 6). Interestingly, in the P6-13 postnatal cortex, locally produced astrocytes are electrically passive, functionally mature and integrated into a network during symmetrical cell division [5]. The differences between this study and ours suggest that neonatal astrocytes differ significantly in their basic electrophysiological properties and the manners in forming cell-to-cell coupling and integration into a syncytial network.

### Neonatal astrocytes are deficient in their K^+^ uptake capacity

In the present study, a substantially low leak K^+^ conductance was detected from neonatal astrocytes. This was indicated by 1) a 6-fold lower inward K^+^ current density than that of outward, and 2) a significantly large and variable *R*_in_ in VRAs (Fig. 3). By altering the K^+^ driving force, we show that the ability of neonatal astrocytes in accumulating intracellular K^+^ concentration is ~50 % less than that of mature astrocytes (Fig. 7).

It should be noted that lack of a maturely established syncytium to achieve a “sustained K^+^ uptake” mode would further undermine the K^+^ uptake and spatial redistribution in the neonatal brain [30]. How the observed difference in K^+^ conductance and gap junction coupling would be etiologically relevant to the neurological disorders in the neonatal brain needs to be further explored.

### Neonatal astrocytes and reactive astrocytes in neurological disorders

Neonatal astrocytes seemingly resemble the reactive astrocytes induced in various pathological conditions in several aspects. First, similar to proliferating neonatal astrocytes, reactive astrocytes reenter the cell cycle for proliferation [57]. Second, the proliferating reactive astrocytes showed virtually no gap junction coupling in dye coupling analysis [57]. Third, neonatal astrocytes predominantly express voltage-gated ion channels, and similar alternation in K^+^ conductance expression has been reported in lesion induced reactive astrocytes [58–60]. In cultured spinal cord astrocytes, K^+^ channels have been demonstrated to play a role in cell cycle progression [61]. Thus, the characteristics of neonatal astrocytes described in this study should serve as an important foundation for further examination into the extent to which reactive astrocytes recapture the features of neonatal astrocytes and their pathological and therapeutic implications [62, 63].

## Conclusions

Neonatal astrocytes homogeneously express a distinct set of rectifying K^+^ conductances, form discrete cell-to-cell coupling and progressively integrate into a syncytial network with age. The passive behavior in some of the neonatal astrocytes is solely attributable to gap junction coupling. The low density expression of the leak K^+^ conductance and lack of a structurally mature syncytium result in a deficient K^+^ homeostasis capacity in neonatal astrocytes. The similarities between neonatal and reactive astrocytes favor a notion that pathological conditions may dedifferentiate mature astrocytes into their neonatal stage in neurological disorders.

### Ethics approval

This study does not need an approval of an ethical committee or consent for publication.

### Open access

This article is distributed under the terms of the Creative Commons Attribution 4.0 International License (http://creativecommons.org/licenses/by/4.0/), which permits unrestricted use, distribution and reproduction in any medium, provided you give appropriate credit to the original author(s) and the source, provide a link to the Creative Commons license, and indicate if changes were made. The Creative Commons Public Domain Dedication waiver (http://creativecommons.org/publicdomain/zero/1.0/) applies to the data made available in this article, unless otherwise stated.

## Abbreviations

[K^+^]_i_: intracellular K^+^ concentration
CC: coupling coefficient
C_M_: membrane capacitance
*C*_reci._: recipient cell
*C*_stim._: stimulated cell
dKO: double gene knockout mouse
DNS: donkey serum
E: embryonic day
*I*_holding_: holding current
*I*K_a_: voltage-gated outward transient K^+^ current
*I*K_d_: voltage-gated outward delayed rectifying K^+^ current
*I*K_in_: inward K^+^ current
*I*Na: voltage-gated inward Na^+^ current
*I-V*: current-to-voltage relationship
K_2p_: two-pore domain K^+^ channel
MFA: meclofenamic acid
OECs: olfactory ensheathing cells
P: postnatal day
PA: passive astrocyte
*R*_a_: access resistance
RI: rectification index
*R*_in_: input resistance
*R*_M_: membrane resistance
SR101: sulforhodamine 101
*V*_COM_: command voltage
*V*_M_: membrane potential
VRA: variably rectifying astrocyte
*V*_rev_: reversal potential
*V*_transjunction_: transjunctional voltage
VZ: ventricular zone
